# A Novel Method for the Combined Photocatalytic Activity Determination and Bandgap Estimation

**DOI:** 10.3390/mps1020022

**Published:** 2018-06-11

**Authors:** Mattia Pierpaoli, Orlando Favoni, Gabriele Fava, Maria Letizia Ruello

**Affiliations:** Department Materials, Environmental Sciences and Urban Planning (SIMAU), Università Politecnica delle Marche, 60131 Ancona, Italy; o.favoni@univpm.it (O.F.); g.fava@univpm.it (G.F.); m.l.ruello@univpm.it (M.L.R.)

**Keywords:** LEDs, photocatalytic reactor, TiO_2_, band gap

## Abstract

The ability of a photocatalyst to degrade a target pollutant is a commonly used method to assess its effectiveness for environmental applications, while ultraviolet-visible (UV-vis) spectroscopy and spectroscopic ellipsometry are conventional techniques for the estimation of a semiconductor band gap. In this work, an array of six light-emitting diodes (LEDs), characterized by different emission peaks between 470–370 nm and absorbed power of 3 W, was implemented into an existing standard testing apparatus for the testing of nitrogen oxides degradation in air. The abatement indexes, obtained under different LEDs irradiation, were firstly compared to the ones determined according the standard and, secondly, correlated with the measured LED emission spectrum, in order to estimate the photocatalyst band gap. Results suggest that this expeditious technique can be easily implemented into existing testing apparatus for the estimation of the band gap and for the appraisal of photocatalytic materials under realistic conditions.

## 1. Introduction

The exponential growth in the number of publications on titanium dioxide photocatalysis, driven mainly by the multidisciplinary nature of the matter, is due to the variety of applications in the environmental and energy fields, including air and water purification systems, self-cleaning surfaces, sterilization, hydrogen evolution, and synthesis of high-added value products.

Ultraviolet (UV)-visible absorption spectra are generally used to estimate the excitation wavelengths, in order to accurately calculate the semiconductor band gap energy, while degradation of methylene blue, nitrogen oxides (NOx), volatile organic compounds (VOCs), among many others (Table 1), under UV or visible irradiation, are the most effective methods to investigate the photocatalytic activity and efficiency.

Our original motivation was to develop a new method to come to terms with both requests: by using a multi-LED (light-emitting diode) reactor, characterized by different selectable emission peaks, we have studied if it is possible to obtain both a good estimation of the photocatalytic activity and the sample bandgap appraisal.

Although titanium dioxide (TiO_2_) is one of the most studied photocatalysts because of the highest efficient photoactivity and stability, the lowest cost and the non-toxicity to humans, its use in environmental applications is limited due the low light energy density and the small amount of solar UV radiation that the TiO_2_ can utilize [1].

Scientific interest is currently mainly addressed in investigating new strategies for enhancing the photocatalytic activity, by increasing surface area and porosity or by incorporation of additional components into the TiO_2_ lattice. Among those, chemical modification is needed to obtain an active photocatalyst in the visible electromagnetic spectrum region.

In this study, four different TiO_2_-based materials have been tested to assess the goodness of the reported method:

- Degussa (Evonik, Essen, Germany) P25 (from now P25) is considered a de facto standard in TiO_2_-photocatalysis because of its relatively high activity. It is well known that the P25 composition is made of anatase and rutile in a variable ratio, typically between 70:30 or 80:20 [1].
- KRONOClean7000 (KRONOS, Dallas, TX, USA) is a carbon-modified commercial anatase mainly addressed for indoor applications, due its visible-light response.
- A silica/titania composite (hSiO_2_/TiO_2_) was prepared, with a hydrothermal method, in our laboratory, with the aim of increasing the surface area of the photocatalyst. The titania content is 7% (*w*/*w*), in the anatase form (Section 3.1).
- The titania-only (hTiO_2_), synthesized under the same conditions, is tested as well for comparison.

Nitrogen oxides (NOx) refer to the couple nitric oxide (NO) and nitrogen dioxide (NO_2_), which is a major criteria in air pollution control, as it is responsible for tropospheric ozone and secondary particulate, and, together with SOx, is the largest contribution for acid rain. 

**Figure 1:**
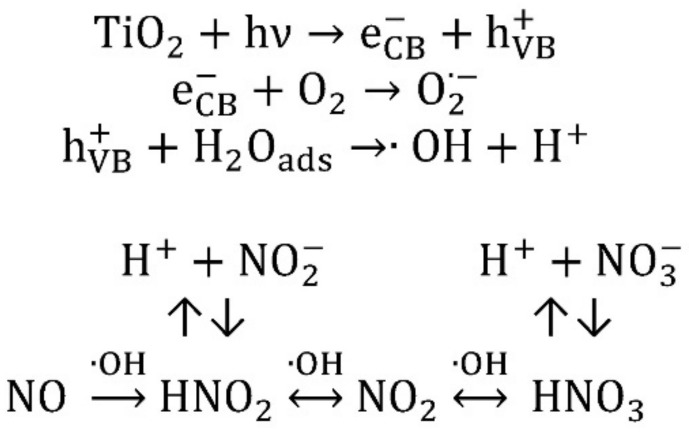
Simplified nitrogen oxides (NOx) reaction mechanism.

Nitrogen oxides degradation is an effective and widely used method to evaluate the photocatalytic activity, as it is possible to evince from Table 1, both because the adsorbability of NO is generally low, both because the oxidative pathway is well reported in literature [2,3,4]. As heterogeneous photocatalysis is a surface phenomenon, the adsorption of NOx over the catalyst is the first step of NOx degradation. Subsequently, when the photocatalyst is irradiated with photons having energy (h*ν*) equal or higher than the bandgap of the semiconductor, it generates an electron–hole pair, which implies the oxidation of NO and the water reduction. A simplified reaction mechanism, proposed by Devahasdin et al. [3], under short space steady state times, is reported in Figure 1.

It is possible to observe that the hydroxyl radical plays a primary role in the overall reaction pathway. Nitrates (NO_3_^−^) produced and accumulated on the surface of the catalyst might act as a physical barrier, inhibiting the photocatalytic process and moving the equilibrium toward the NO_2_.

The most common sources of UV are low, medium, and high-pressure mercury arc lamps, which show the disadvantages associated with their fragility and hazardous mercury content, which relates also to their disposal. The low efficiency, due the high heat generated, and their relatively short working lifespan (500−2000 h) make them unsuitable for full-scale applications. On the other way, LEDs have already been used for many photocatalytic applications, due to their low cost, longer lifespan than conventional light sources, low energy consumption and to their well-defined emission peak, which makes unnecessary the use of a cutoff filter [5].

Levine et al. [6] demonstrated that the UV-A LED is a viable alternative to the Hg-vapor lamps if the design of the LED arrays is improved to increase the lighting uniformity and the results proved that LEDs are a viable photon source both in terms of reaction quantum efficiency and wall plug efficiency. For this reason, modeling and computation fluid dynamic became important studies [7].

Wang et al. [8] used four different LED strips emitting blue, green, yellow and white lights to activate a C–N co-doped TiO_2_, and they found a decreasing bisphenol A removal efficiency by increasing the wavelength of the LED.

An interesting application of LED was made by Korovin et al. [9], in which was found that the usage of controlled periodic illumination increases quantum efficiency as duty cycle approaches small values. Moreover, such experiment is a powerful tool for direct evaluation of lifetimes of reactive intermediates.

A few authors have already implemented LEDs as a luminous source to activate the photocatalysts, in order to study of the photocatalytic activity of such materials towards the degradation of gaseous pollutants [6,7,9,10], water pollutants [8,11,12,13], dyes [14] and bacteria inactivation [15,16]. As the choice of a suitable light source is a fundamental choice, the use of an array of LEDs, characterized by different emitted wavelengths, can provide more information on the photocatalysts under examination. For these reasons, six LEDs, characterized by different emission peaks, were used in this work to activate the photocatalysts and to provide a rough estimation of the photocatalyst bandgap. According to the current knowledge of the authors, this is the first attempt to estimate the bandgap of different photocatalysts, through the direct measurement of a target pollutant degradation under different lighting conditions.

## 2. Materials and Methods

### 2.1. Characterization of TiO_2_-Based Materials

Ultraviolet–visible (UV-Vis) diffuse reflectance spectroscopy is one of the most employed method for the determination of the band gap energy of semiconductors and amorphous solid material.

Through the absorption spectra, estimated by the Kubelka-Munk method, UV-Vis spectroscopy provides information about the electronic transitions of the different orbitals of a solid. In this study, a Jasco V-670 spectrometer (JASCO Germany GmbH, Pfungstadt, Germany), equipped with a Jasco ILN-725 integrating sphere (JASCO Germany GmbH, Pfungstadt, Germany), has been used to measure the diffuse reflectance of the samples, in the range of 200–800 nm (data interval = 0.5 nm, UV-vis bandwidth = 1.0, scan speed = 200 nm/min). Bandgap energy has been estimated by performing the Kubelka-Munch transformation.

The powder X-ray diffraction (XRD) patterns were recorded on was performed with a Philips PW 1730 diffractometer (Philips, Eindhoven, Netherlands) (CuK radiation and 0.02 2*θ* s^−1^ scanning rate) operating between 3 and 50 2*θ* and equipped with software for the spectra evaluation.

### 2.2. Nitrogen Oxides Apparatus

**Figure 2:**
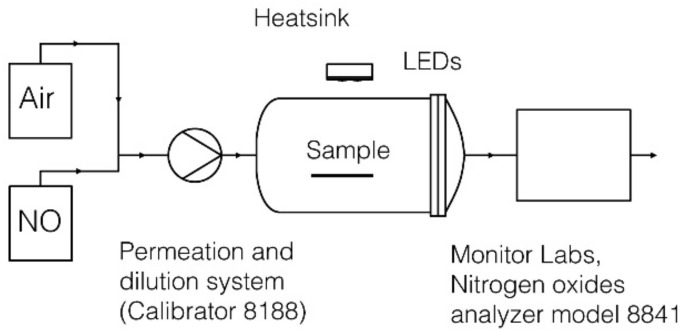
Apparatus schematic. Abbreviation: LED, light-emitting diode.

The apparatus, the test conditions and the reactor were adopted by the Italian standard UNI 11247, where a continuous flow test method is used for the determination of the degradation of nitrogen oxides in the air by inorganic photocatalytic materials. Figure 2 shows the experimental apparatus. 

The NOx flux inside the reactor is provided by a NOx tank (499 ppb NO) (SAPIO S.r.l., Monza, Italy) and it is kept constant with a dilution system (Calibrator 8188, Rancon Instruments s.p.a., Milan, Italy). Dilution is obtained by mixing with atmospheric air at room temperature (27 ± 2 °C) and relative humidity between 40–50%. Outlet gaseous NO and NO_2_ concentrations were continuously monitored by a chemiluminescence NOx analyzer (Nitrogen oxides analyzer model 8841, Monitor Labs, Englewood, CO, USA).

The photoreactor consists of a 3 L Pyrex glass cylinder, LEDs are located at the center of the reactor, over the sample, outside the reactor. The samples examinations are positioned inside at the center of the reactor.

Colloidal samples of various TiO_2_-based powders were prepared by sonication of an aqueous slurry for one hour, followed by concentration on a glass surface by slow evaporation under an infrared (IR) lamp. The dried samples were stored in a sealed chamber and exposed one hour to laboratory environments, prior the test. 

### 2.3. LED Characterization

Light-emitting diodes were purchased from Shenzhen Chundaxin Photoelectric Co. (Shenzhen, China) and they were chosen for their different emission peak; their characteristics are reported in Table 2.

Light-emitting diode spectrums were measured in the 200–800 nm range by using a spectrometer (model CAS 120, Instrument Systems, Munich, Germany). The CAS 120 is equipped with a crossed Czerny-Turner spectrograph and an array detector. The spectral resolution is 2.7 nm, the data point interval is 0.35 nm, the wavelength accuracy is ±0.3 nm, and the integration time is 60–870 ms. Spectral irradiance ($E_{e,\lambda }$) was measured at three distances (*d*) and it was found the following inverse-squared relation:

(1) $$E_{e,\lambda }=\alpha \frac{1}{d^{2}}.$$

Spectrum peaks and widths were calculated by approximating spectra to normal distributions:

(2) $$E_{e,\lambda }=\frac{1}{\sqrt{2\pi \sigma^{2}}}\exp\left(-\frac{1}{2}{\left(\frac{\lambda -\mu }{\sigma }\right)}^{2}\right),$$

where μ is the wavelength at which the spectrum is centered (nm) and $\sigma^{2}$ is the variance.

Data fitting was performed with MATLAB data fit tool. For sake of simplicity, Table 2 reports only one set of measurements, at a given distance, with the corresponding data fitting results and goodness.

**Figure 3:**
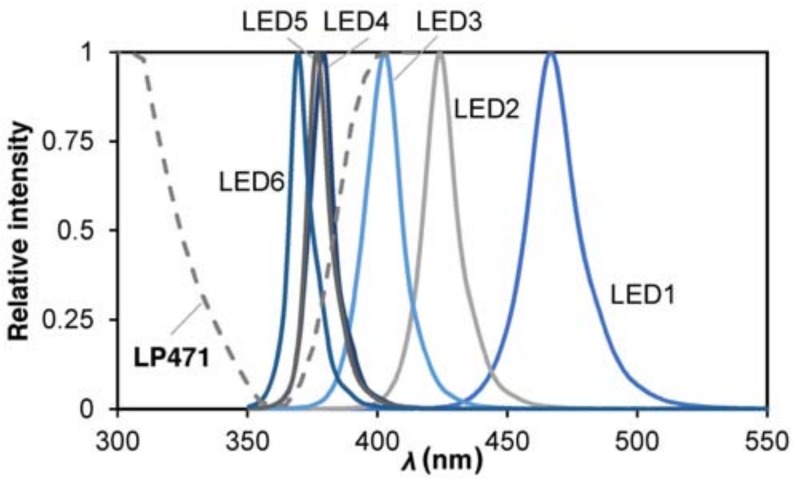
LEDs emission spectra.

Measured LEDs relative intensity of emission spectra are reported in Figure 3. 

This combination of LEDs was chosen to point to the study in the 420–365 nm region, as showed by the overlapping region of the different spectra. It can be noticed how the emission peaks differs from the central value of the declared interval. Irradiance was also measured with a photoradiometer (Delta Ohm, HD2102.2, Padua, Italy) with a probe centered in the field of the UV-A with a resolution of 0.001 W·m^−2^ (LP471), inside the reactor, for keeping into account the effect of the borosilicate glass reactor. This optical window is reported in Figure 3 by the dotted line.

The intensity of the photon energy ($E_{\lambda }$) provided by the different LEDs lights was calculated from the inverse relationship:

(3) $$E=\frac{hc}{\lambda },$$

where *h* is Planck’s constant and *c* is the speed of light. The values of the used constants are h = 6.626 × 10^−34^ J·s and c = 2.998 × 10^8^ m/s.

### 2.4. Test Procedure Description

**Figure 4:**
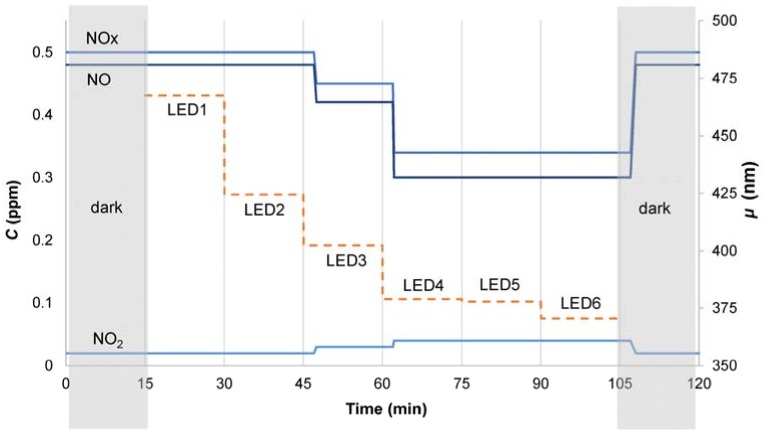
NO and NO_2_ concentrations during test condition under different LED illumination.

Once the sample is placed inside the reactor, and the inlet NOx gas concentration is stationary, the first LED, characterized by the highest *μ*, is turned on. After 15 min, it is turned off and the second LED is turned on for other 15 min. This procedure is repeated until all the six LEDs are exposed; the NOx and NO concentrations are monitored during the whole test. A generic schematic of the test is shown in Figure 4.

It is possible to observe in Figure 4 the time shift between the moment at which the LED is turned on and the beginning of the decrease of the NOx concentration, a lag time that indicates the hydraulic residence time of the photocatalytic reactor.

NO activity ($A_{\mathrm{NO}}$) is an index to describe the photocatalytic activity toward the degradation of NO, in which $C_{\mathrm{NO}}^{\mathrm{dark}}$ and $C_{\mathrm{NO}}^{\mathrm{light}}$ are respectively the averaged concentrations of NO during dark and UV conditions, for each LED:

(4) $$A_{\mathrm{NO}}=\frac{C_{\mathrm{NO}}^{\mathrm{dark}}-C_{\mathrm{NO}}^{\mathrm{light}}}{C_{\mathrm{NO}}^{\mathrm{dark}}}.$$

Similarly, the difference between the NO_2_ concentration under light and dark conditions, normalized on the NO concentration, can be considered as an index related to the unwanted NO_2_ selectivity. As $C_{\mathrm{NO}2}^{\mathrm{light}}$ is greater than $C_{\mathrm{NO}2}^{\mathrm{dark}}$, the minus in front of the equation is to maintain positive sign to the index:

(5) $$A_{\mathrm{NO}2}=-\frac{C_{\mathrm{NO}2}^{\mathrm{dark}}-C_{\mathrm{NO}2}^{\mathrm{light}}}{C_{\mathrm{NO}}^{\mathrm{dark}}}.$$

### 2.5. Data Elaboration

By comparing the $A_{\mathrm{NO}}$ index, it was possible to individuate the wavelength at which the photocatalyst activates, so to estimate the bandgap. Two methods are reported:

A first more intuitive approach is based on a simply observation at which LED interval the photocatalyst activation occurs. An activity ($A_{\mathrm{NO}}$) greater than 0.05 is assumed as a threshold between the minimal observable activity and the background noise; this method however only provides an interval of reference, which strictly depends on the chosen LEDs.

The second method proposed provides a more accurate estimate of the activation energy. A detailed explanations of the method adopted is described in the following. 

Since the LED emission spectrum is well represented by a normal distribution, the cumulative distribution function is given by the equation:

(6) $$E_{e,\bar{\lambda }}\;=\frac{1}{2}\left(1+\mathrm{erf}\left(\frac{\bar{\lambda }-\mu }{\sigma \sqrt{2}}\right)\right).$$

**Figure 5:**
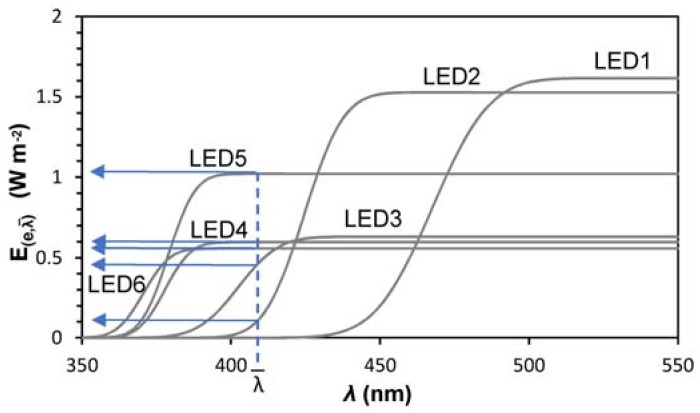
Cumulative distribution function adopted for the representation of used LEDs.

$E_{e,\bar{\lambda }}$ represents the total irradiance for λ < $\;\bar{\lambda }$, in which $\;\bar{\lambda }$ is the highest wavelength, at which corresponds the minimum energy necessary to activate the photocatalyst. For ease of interpretation, these functions are plotted in Figure 5. 

Assuming that a generic photocatalyst, having a band gap of *E*_0_, is activated by an electromagnetic radiation having a wavelength at $\lambda <\frac{hc}{E_{0}}$, it will exhibit photocatalytic activity A_NO_, only when radiated by LEDs 2, 3, 4, 5 and 6. Moreover, the relation between photon flow (light irradiance) and photocatalytic activity, for $E_{e}$ < 1 Wm^−2^, expressed as NO oxidation rate, can be considered linearly dependent [16,17].

**Figure 6:**
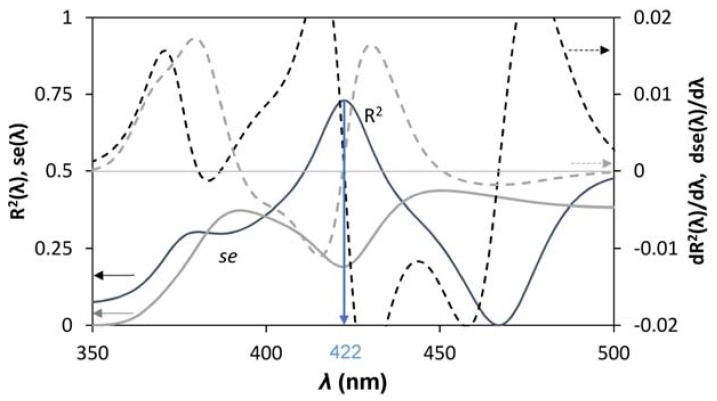
Determination of the best correlation between *E*_(*e*,*λ*)_ and A_NO_ by computing the coefficient of determination (black solid line), and minimizing the standard error (gray solid line). Minimums and maximums are highlighted by the first derivative (dotted lines).

By computing the goodness of the linear fit between $E_{e,\bar{\lambda }}$ and A_NO_, for A_NO_ > 0, in the interval $350<\bar{\lambda }<500$ (nm), it is possible to estimate the value of λ. The goodness of the fit was evaluated by mean of maximizing the coefficient of determination, R^2^, and minimizing standard error, *se*. To highlight the maximum and minimum of the functions, the first derivative was computed. An example is reported in Figure 6.

**Figure 7:**
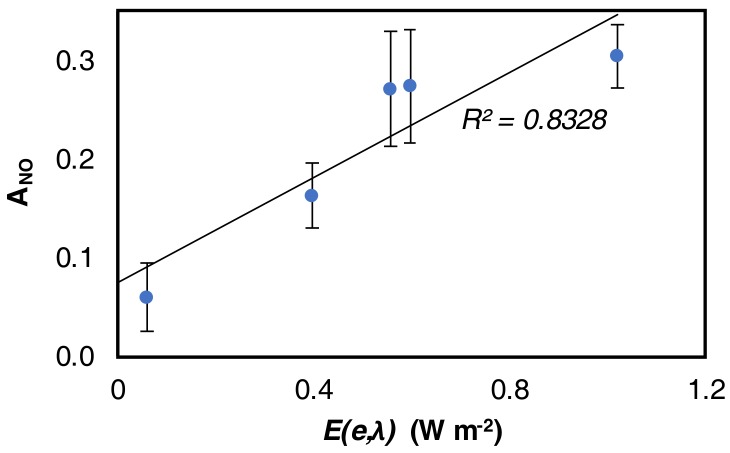
Correlation between A_NO_ and *E*.

Once λ has been estimated, it is possible to plot the relation between the two variables in order to evaluate the fit (Figure 7).

## 3. Results

### 3.1. Characterization of TiO_2_-Based Materials

The band gaps optically obtained by plotting (Kh*ν*)^1/2^ versus the energy of absorbed light are approximately 3.05 and 3.25 eV, respectively for the P25 and the two titania synthesized by the hydrothermal method. The KRONOClean shows a bandgap of 2.32 eV.

**Figure 8:**
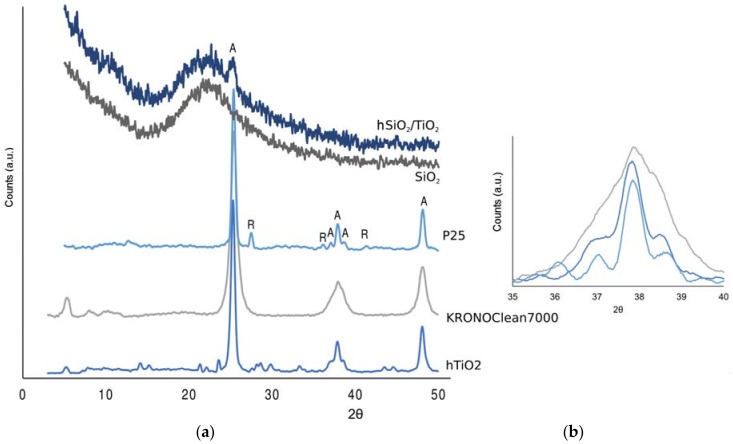
(**a**) X-ray diffraction (XRD) patterns of (from bottom to top) hTiO_2_, KRONOClean7000, P25, SiO_2_, hSiO_2_/TiO_2._ (**b**) XRD for the three distinct peaks characteristic of anatase.

The X-ray diffraction pattern of the different titania samples is shown in Figure 8. 

In all sample, TiO_2_ is present in the anatase form, as indicated by the A-peaks, while only the P25 shows also traces of rutile. In-lab prepared hTiO_2_ shows some impurities, but no rutile as no peaks are reported at 2*θ* equal to 35.97 and 41.11.

The possibility of distinguishing the three distinct peaks (2*θ* = 36.95, 37.79, 38.57) can be related to the crystallinity and the calcination temperature of the material [18]. This is in agreement with the KRONOClean7000 wide peak, which can indicate a lower calcination temperature.

### 3.2. Nitrogen Oxides Abatement

Nitrogen oxides removal indexes, obtained according to the Italian standard UNI11247, have been determined equals to 36%, 41%, 43%, 46%, respectively for hTiO_2_, hSiO_2_/TiO_2_, P25, KRONOClean7000^®^ samples.

Nitrogen oxides removal indexes, obtained according to the procedure described in Section 2.4, are reported in Table 3. It is found that:

- generally, the amount of NO_2_ generated is higher at higher wavelengths;
- generally, to a higher NO activity corresponds a higher production of NO_2_

**Figure 9:**
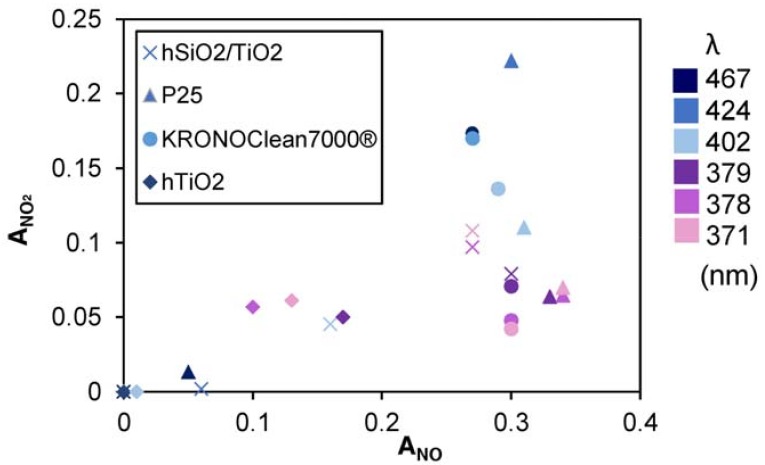
Activity indexes grouped by sample and UV light.

By plotting values reported in Table 3 and Figure 9, it is possible to have a straightforward view of the material goodness: the score shape describe the sample and the color label the LED used. High A_NO_ scores and low A_NO2_ scores are desirable.

### 3.3. Bandgap Estimation

Results of the bandgap estimation, by minimizing the standard error and maximizing the coefficient of determination, are reported in Table 4. Estimated bandgap energies, ($E_{\lambda ,ext}$) fall in the determined activation ranges and they are compatible with the values obtained through UV-vis spectroscopy or reported in the literature ($E_{\lambda }$).

## 4. Discussion

### 4.1. The Method

**Figure 10:**
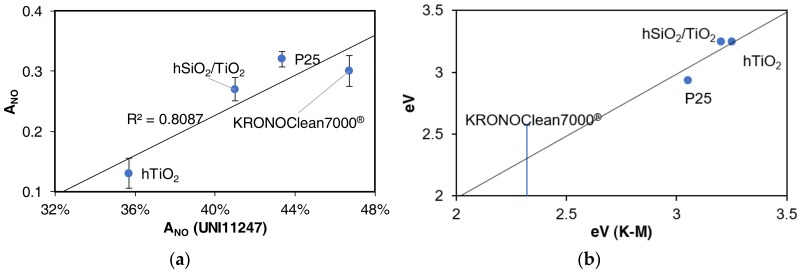
(**a**) Activity indexes grouped by sample and UV light, (**b**) relation between the band gap estimated by diffuse UV-visible spectroscopy and the proposed method.

Figure 10 shows the comparison between the proposed method and the NOx standard (a) and to the Kubelka-Munk estimation (b). By increasing the number of LEDs having emission peak close to the correspondent expected photocatalyst bandgap, it is possible to better estimate its value. For instance, as KRONOClean7000^®^ exhibits photocatalytic activity since the first LED is turned on, it is not possible to individuate the end of the activation interval, while the method provides a good estimation for hSiO_2_/TiO_2_, hTiO_2_, P25 with, respectively, 4, 4 and 5 different LED emission spectrum possess enough energy to activate the samples.

The imperfect correspondence between the two procedures, reported in Figure 10a, is due to the fact that two different samples were prepared for the two different tests.

### 4.2. NO Activity

It is well known that anatase shows a higher photocatalytic activity [19,20] than rutile. At 422 nm (2.94 eV) P25 shows photocatalytic activity, as rutile is reported to activate at 3.0 eV [21,22], and it slightly increases with lowering the wavelength.

The silica/titania composite (hSiO_2_/TiO_2_) and the single titania (hTiO_2_) exhibit similar photocatalytic activity. Despite the amorphous nature of the silica in the composite, XRD diffractogram indicates the presence of anatase, showed by the peak at 2*θ* = 25.32.

KRONOClean7000 is a C-modified anatase and it was found to be active already at the first LED irradiation, with *λ* > 467 nm.

### 4.3. NO_2_ Selectivity

Selectivity is an important parameter to take into account. In this case, the production of NO_2_, as an unwanted product, was monitored. Visible-light photocatalysts have been under great observation because of their possible application in indoor conditions; however, their effective beneficial provision must be questioned. Bloh et al. [23] arbitrarily assigned a relative toxicity ratio of 1:3, for NO and NO_2_. For this reason, systems characterized by high activity, but low selectivity into final products (nitrates) could potentially increase the indoor air toxicity by the formation of NO_2_. In fact, the application of photocatalytic materials in cement matrices can occur by the combined use of materials with high adsorbent capacity [24].

The commercial KRONOClean7000, despite its low bandgap making it suitable for indoor applications, exhibits a high production of NO_2_ (17.36%) at high wavelengths. At lower wavelengths (*λ* < 379 nm) unwanted NO_2_ selectivity decreases to 4.2%. A similar behavior belongs to the P25, with the only difference that it activates between 424 and 427 nm. This can be due the typical ternary composition of P25, where anatase, rutile, and amorphous TiO_2_ are present in an average ratio of 78:14:8 [1] and the higher production of NO_2_, as well the lower mineralization of organic compounds, it is linked to the presence of rutile [1,24]. However, the increased selectivity into NO_2_ by higher wavelengths was confirmed also by Tseng et al. [25].

For the hSiO_2_/TiO_2_ sample, where TiO_2_ in anatase form is deposited over a silica substrate, NO_2_ production is lower (11%); similarly for the hTiO_2_ sample obtained through the same hydrothermal method, but without the silica substrate, which shows a lower production of NO_2_. However, this aspect is easily explained by the fact that the efficiency is also lower.

## 5. Conclusions

The bandgap energy and the photocatalytic activity of four TiO_2_-based material have been jointly estimated by a novel approach based on the effective degradation of NOx, by using a multi-spectrum array of LEDs. The procedure can be easily implemented on an existing testing apparatus, moreover, LEDs are cheap and it is not necessary to shield the light source because of the well-defined emission spectrum.

It is noticed that this multi-UV testing method can provide useful indications either on the activity of the photocatalytic material under different lighting conditions, either on the effectiveness of such material against unwanted by-products.

The suggested method forms a direct assessment of the photocatalytic activity under plausible conditions of irradiance (and pollutant concentration).

## Figures and Tables

**Table 1:** Reference standards for the determination of the photocatalytic activity, grouped by target compound.

Target Pollutant	Type of Irradiation	
	UV	Visible
NOx	UNI 11247	DIS 17168-1
	ISO 22197-1	
	DIN CEN/TS 16980-1	
	JIS R 1701-1	
Acetaldehyde	ISO 22197-2	DIS 17168-2
	JIS R 1701-2	CD 19652
Toluene	ISO 22197-3	DIS 17168-3
	JIS R 1701-3	
Formaldehyde	ISO 22197-4	DIS 17168-4
	JIS R 1701-4	ISO 18560-1
Methyl mercaptan	ISO 22197-5	DIS 17168-5
	JIS R 1701-5	

Abbreviation: UV, ultraviolet.

**Table 2:** Light-emitting diodes (LEDs) characteristics.

	µ, Spectrum Peak (nm)		σ^2^, Variance (nm^2^)	$E_{e,\lambda }$ (W m^−2^ nm^−1^)	R^2^
	Declared *	Effective			
LED 1	blue	467.5	15.2	1.11	0.9860
LED 2	420–430	424.4	10.5	1.10	0.9845
LED 3	400–410	402.4	10.8	0.46	0.9927
LED 4	385–390	378.9	7.2	0.74	0.9848
LED 5	375–380	377.8	6.9	0.43	0.9782
LED 6	365–370	370.6	7.0	0.40	0.9726

* Declared by the producer.

**Table 3:** Calculated photocatalytic NOx abatement indexes.

LED		hSiO_2_/TiO_2_		P25		KRONOClean7000		hTiO_2_	
Name	Peak (nm)	A_NO_	A_NO_	A_NO_	A_NO2_	A_NO_	A_NO2_	A_NO_	A_NO2_
LED 1	467	0.00	0.00	0.05	0.01	0.27	0.17	0.00	0.00
LED 2	424	0.06	0.00	0.30	0.22	0.27	0.17	0.00	0.00
LED 3	402	0.16	0.05	0.31	0.11	0.29	0.14	0.01	0.00
LED 4	379	0.30	0.08	0.33	0.06	0.30	0.07	0.17	0.05
LED 5	378	0.27	0.10	0.34	0.06	0.30	0.05	0.10	0.06
LED 6	371	0.27	0.11	0.34	0.07	0.30	0.04	0.13	0.06

**Table 4:** Results from the bandgap estimation.

Sample	Activation Range	$\lambda_{ext}$ (nm)	$E_{\lambda ,ext}$ (eV)	$E_{\lambda }$ (eV)	Ref.
P25	LED 1 < λ < LED 2	422	2.94	3.05	
KRONOClean7000^®^	λ > LED 1	>467	<2.65	2.32	[17]
hSiO_2_/TiO_2_	LED 2 < λ < LED 3	382	3.25	3.20	
hTiO_2_	LED 3 < λ < LED 4	382	3.25	3.25	
Anatase				3.2	[18]
Rutile				3.0	[18]
